# Evaluation of multi-assay algorithms for identifying individuals with recent HIV infection: HPTN 071 (PopART)

**DOI:** 10.1371/journal.pone.0258644

**Published:** 2021-12-17

**Authors:** Wendy Grant-McAuley, Ethan Klock, Oliver Laeyendecker, Estelle Piwowar-Manning, Ethan Wilson, William Clarke, Autumn Breaud, Ayana Moore, Helen Ayles, Barry Kosloff, Kwame Shanaube, Peter Bock, Nomtha Mandla, Anneen van Deventer, Sarah Fidler, Deborah Donnell, Richard Hayes, Susan H. Eshleman, for the HPTN 071 (PopART) Study Team

**Affiliations:** 1 Department of Pathology, Johns Hopkins University School of Medicine, Baltimore, Maryland, United States of America; 2 Department of Medicine, Johns Hopkins University School of Medicine, Baltimore, Maryland, United States of America; 3 Division of Intramural Research, National Institute of Allergy and Infectious Diseases, National Institutes of Health, Rockville, Maryland, United States of America; 4 Fred Hutchinson Cancer Research Center, Seattle, Washington, United States of America; 5 FHI360, Durham, North Carolina, United States of America; 6 Zambart, University of Zambia School of Medicine, Lusaka, Zambia; 7 Clinical Research Department, London School of Hygiene and Tropical Medicine, London, United Kingdom; 8 Desmond Tutu TB Center, Department of Paediatrics and Child Health, Stellenbosch University, Western Cape, South Africa; 9 Department of Infectious Disease, Imperial College London, London, United Kingdom; 10 Department of Infectious Disease Epidemiology, London School of Hygiene and Tropical Medicine, London, United Kingdom; Tel Aviv University Sackler Faculty of Medicine, ISRAEL

## Abstract

**Background:**

Assays and multi-assay algorithms (MAAs) have been developed for population-level cross-sectional HIV incidence estimation. These algorithms use a combination of serologic and/or non-serologic biomarkers to assess the duration of infection. We evaluated the performance of four MAAs for individual-level recency assessments.

**Methods:**

Samples were obtained from 220 seroconverters (infected <1 year) and 4,396 non-seroconverters (infected >1 year) enrolled in an HIV prevention trial (HPTN 071 [PopART]); 28.6% of the seroconverters and 73.4% of the non-seroconverters had HIV viral loads ≤400 copies/mL. Samples were tested with two laboratory-based assays (LAg-Avidity, JHU BioRad-Avidity) and a point-of-care assay (rapid LAg). The four MAAs included different combinations of these assays and HIV viral load. Seroconverters on antiretroviral treatment (ART) were identified using a qualitative multi-drug assay.

**Results:**

The MAAs identified between 54 and 100 (25% to 46%) of the seroconverters as recently-infected. The false recent rate of the MAAs for infections >2 years duration ranged from 0.2%-1.3%. The MAAs classified different overlapping groups of individuals as recent vs. non-recent. Only 32 (15%) of the 220 seroconverters were classified as recent by all four MAAs. Viral suppression impacted the performance of the two LAg-based assays. LAg-Avidity assay values were also lower for seroconverters who were virally suppressed on ART compared to those with natural viral suppression.

**Conclusions:**

The four MAAs evaluated varied in sensitivity and specificity for identifying persons infected <1 year as recently infected and classified different groups of seroconverters as recently infected. Sensitivity was low for all four MAAs. These performance issues should be considered if these methods are used for individual-level recency assessments.

## Introduction

Characterization of individuals with recent HIV infection can provide important information about the state of the HIV/AIDS epidemic, inform the impact of interventions for HIV prevention, and help characterize populations at increased risk of infection [1]. Recently-infected persons may also be more likely to transmit HIV to others, especially if they are not aware of their infection status and are not yet on antiretroviral treatment (ART) [2]. Multi-assay algorithms (MAAs, also referred to as Recent Infection Test Algorithms [RITAs]) have been developed for estimating population-level incidence in cross-sectional surveys; these algorithms use two or more assays to classify samples as “recent” vs. “non-recent” [3–9]. MAAs have also been used in research studies to identify and characterize individuals with recent HIV infection [10–13]. Other studies have noted the potential to use MAAs in real-time to inform persons of their recency status [14, 15], to identify patients with recent infection for clinical purposes [16], or to inform epidemiologic surveillance studies and public health responses to the HIV epidemic [17, 18]. However, there are limited data assessing the performance of MAAs for individual-level recency assessments [19].

Most multi-assay algorithms (MAAs) used for cross-sectional HIV incidence estimation include a combination of serologic and non-serologic assays [5–9]. The limiting antigen avidity enzyme immunoassay (LAg-Avidity assay) is the most widely used serologic assay for HIV incidence estimation [20, 21]. This assay was developed by the United States (US) Centers for Disease Control (CDC) and is commercially available. A point-of-care, qualitative LAg-Avidity assay is also available. These assays are intended for research use only and are not cleared by the US Food and Drug Administration (FDA) for clinical use [22, 23]. The most widely used MAA for cross-sectional incidence estimation includes the LAg-Avidity assay plus HIV viral load [20, 24]. In this MAA, low HIV viral load is assumed to be due to ART; persons with low viral loads are classified as not recently infected [20, 24]. Individuals who start ART early in infection and achieve viral suppression may be misclassified using this approach [25]. This type of misclassification is likely to increase over time, since ART is now recommended for all HIV-infected persons, regardless of CD4 cell count [26].

Viral suppression can also impact the accuracy of individual serologic incidence assays by altering the antibody response to HIV infection [27–30]. Individuals who are virally suppressed on ART have low levels of circulating virus. In these persons, the level or avidity of anti-HIV antibodies may be reduced [27], which can impact the performance of serologic incidence assays [28–30]. Down-regulation of anti-HIV antibodies has also been observed in HIV controllers, who are virally suppressed in the absence of ART [27]. We recently demonstrated that anti-HIV antibody responses are different in persons with natural vs. ART-induced viral suppression [31]. It is not known if serologic incidence assays perform differently in individuals with natural vs. ART-induced viral suppression.

In this report, we analyzed the performance of MAAs for identifying individuals with recent infection. This study was conducted using samples and data from the HIV Prevention Trials Network 071 trial (HPTN 071, PopART) [32]. This community-randomized trial was conducted in Zambia and South Africa and evaluated the impact of a combination prevention intervention that included universal testing and treatment on HIV incidence [32]. Early ART initiation could potentially confound performance of serologic incidence assays if a substantial portion of individuals with recent infection are virally suppressed. HPTN 071 (PopART) enrolled a randomly-selected Population Cohort (PC) of >48,000 participants who were followed for up to three years [32]. High rates of viral suppression were achieved in the PC after two years of study intervention [32]. We previously demonstrated that two MAAs provided accurate point estimates of HIV incidence in this cohort [33]. In this report, we evaluated whether four MAAs developed for population-level cross-sectional HIV incidence estimation could accurately identify individuals in HPTN 071 (PopART) who were infected <1 year. This cohort included many individuals who were virally suppressed early in infection. This allowed us to extend our analysis to include a comparison of the impact of natural vs. ART-induced viral suppression on the performance of serologic incidence assays.

## Methods

### Samples used for analysis

**Fig 1:**
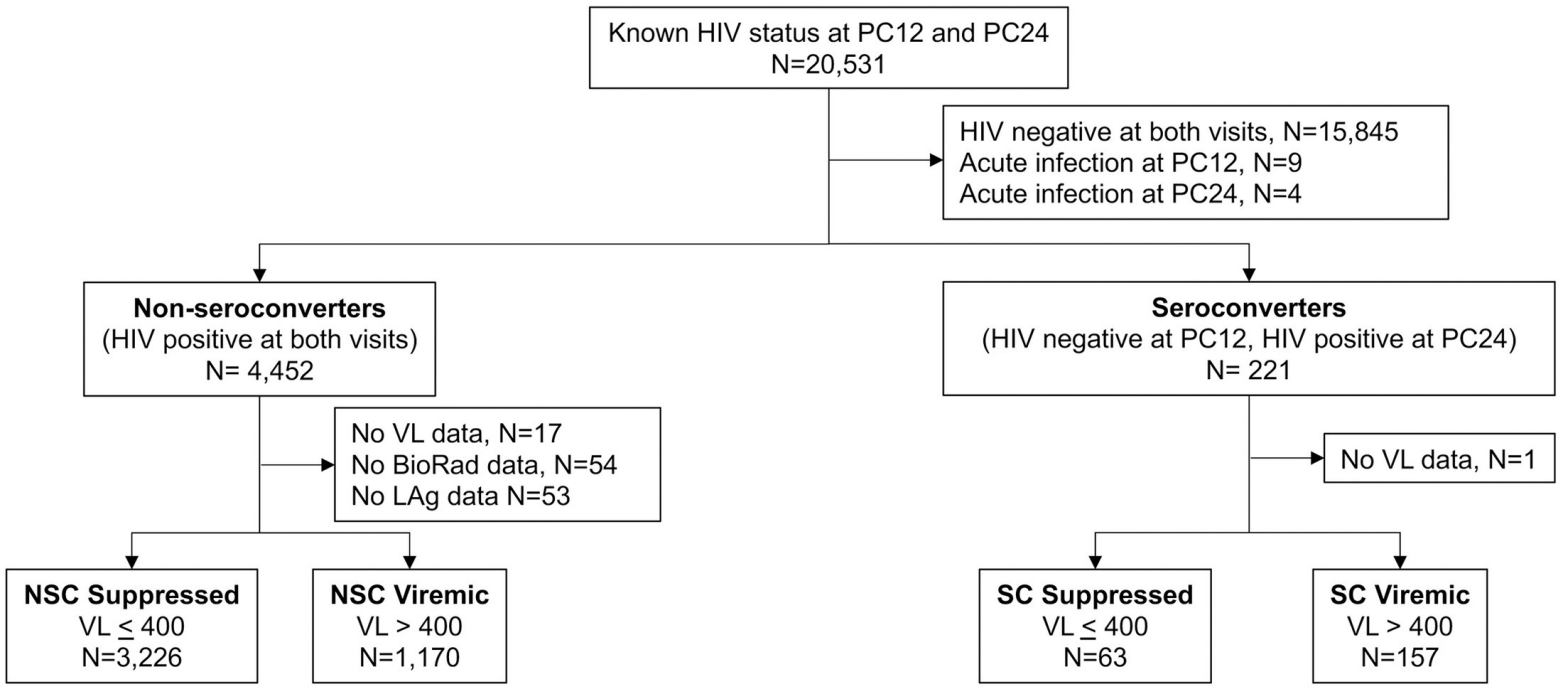
Samples included in the analysis. The figure shows the process used to select samples for analysis. Abbreviations: N: number; PC: Population Cohort; PC12: study visit in HPTN 071 (PopART) after 12 months of study intervention; PC24: study visit in HPTN 071 (PopART) after 24 months of study intervention; VL: viral load; BioRad: JHU BioRad-Avidity assay; LAg: LAg-Avidity assay; NSC: non-seroconverter; SC: seroconverter.

This study was conducted using samples and data from the HPTN 071 (PopART) trial (NCT 019000977) carried out in 21 study communities in Zambia and South Africa. Most HIV infections in the study countries are HIV subtype C. Samples were collected from participants in the PC at annual visits, referred to as PC0, PC12, PC24, and PC36. HIV status was determined for PC participants at each study visit at the HPTN Laboratory Center [34]. This report is based on analysis of samples collected at the PC24 visit. The analysis was limited to participants who had HIV status determined at the PC12 and PC24 visits (Fig 1); participants with acute HIV infection (RNA positive, antibody negative) at either of these visits were excluded from analysis. The analyses in this study included two groups of participants: (1) participants who were HIV negative at PC12 and HIV positive at PC24 (infected <1 year, seroconverters), and (2) participants who were HIV-positive at both the PC12 and PC24 visits (infected >1 year, non-seroconverters). Median duration between the PC12 and PC24 visits was 391 days (interquartile range [IQR]: 345, 436) for the seroconverter group and 382 days (IQR: 341, 421) for the non-seroconverter group. HIV status from the PC0 visit was available for a subset of the participants in the non-seroconverter group; these data were used to identify those infected for >2 years.

### Laboratory methods

The testing described in this report was performed at the HPTN Laboratory Center (Baltimore, MD). Recency was assessed using four MAAs that included two or more assays (Table 1). These four MAAs were developed for population-level cross-sectional HIV incidence estimation. The HIV-1 Limiting Antigen (LAg)-Avidity EIA (Sedia Biosciences Corporation, Beaverton, OR; LAg-Avidity assay) was performed according to manufacturer’s instructions. The JHU BioRad-Avidity assay was performed as previously described [35]; this assay is based on the Genetic Systems 1/2 + O ELISA (Bio-Rad Laboratories, Redmond, WA). The rapid LAg assay (Asanté HIV-1 Rapid Recency assay, Sedia Biosciences Corporation, Beaverton, OR) was performed according to manufacturer’s instructions; results for all samples were read manually by a single laboratory technician. Samples where the long-term infected band was not detected were categorized as recent. Viral load testing was performed previously using the RealTime HIV-1 assay (Abbott Molecular, Des Plaines, IL) with a validated dilution procedure (limit of quantification: 400 copies/mL) [32]. Antiretroviral (ARV) drug testing was performed using an assay that detects 22 drugs in five drug classes [36]. Detection of one or more ARV drugs was considered to be indicative of ART.

**Table 1:** Multi-assay algorithms evaluated in this report.

MAA	Laboratory results used to identify recent infections	Mean window period (days)
LAg MAA^a^	LAg OD-n <1.5 plus viral load >1,000 copies/mL	130
MAA-C^b^	LAg OD-n <2.8 plus BioRad avidity index <95% plus viral load >400 copies/mL	248
Rapid MAA^c^	Rapid LAg “recent” plus viral load >1,000 copies/mL	180
Alternate MAA^d^	BioRad avidity index <40% plus LAg OD-n <2.8	119

The table shows the assays and cutoffs included in the four multi-assay algorithms (MAAs) evaluated in this report. These MAAs were developed for cross-sectional incidence HIV estimation, not for individual-level recency assessments. The mean window period indicates the average time after infection that persons are classified as recent.

^a^ The LAg MAA is widely used for HIV incidence estimation [22].

^b^ MAA-C has been used for HIV incidence estimation in prior studies [9].

^c^ The Rapid MAA includes a point-of-care serologic assay; this makes it easier to deploy the MAA in clinic or field settings [23].

^d^ The Alternate MAA does not include viral load as a biomarker; this may be an advantage for assessing incidence and recency in settings where a substantial portion of individuals with recent infection are virally suppressed on antitretroviral treatment [4].

Abbreviations: LAg: HIV-1 Limiting Antigen (LAg)-Avidity EIA (Sedia Biosciences Corporation); BioRad: JHU BioRad-Avidity assay (based on the Genetic Systems 1/2 + O ELISA, Bio-Rad Laboratories). Rapid LAg: Asanté HIV-1 Rapid Recency assay (Sedia Biosciences); OD-n: normalized optical density units; mL: milliliter.

### Statistical methods

Sensitivity and specificity for classifying persons infected <1 year as recently infected were calculated using seroconversion status as the gold standard for classifying participants as recent vs. non-recent. False recent rates (FRRs) were calculated as the percentage of persons infected >2 years who were classified as recently infected [37]. Univariate analyses were performed using the t-test, chi-square test, or Fisher’s exact test. Multivariate analysis was performed using linear regression analysis for continuous output variables and logistic regression analysis for binary output variables. The final multivariate regression models were determined using backward stepwise procedures. Statistical analysis was performed using R version 4.0.2 [38], the MASS package [39], and the epiDisplay package [40].

### Informed consent

Written informed consent was obtained from participants prior to enrollment in HPTN 071 (PopART). The HPTN 071 (PopART) study was approved by the institutional review board and ethics committees at the London School of Hygiene and Tropical Medicine, the University of Zambia, and Stellenbosch University.

## Results

### Study cohort

In HPTN 071 (PopART), 20,531 participants had HIV status determined at both the PC12 and PC24 visits (Fig 1). This included 4,452 participants who were HIV positive at both visits (non-serconverters) and 221 participants who were HIV negative at PC12 and HIV positive at PC24 (seroconverters). Fifty-six participants were excluded from the non-seroconverter group because they were missing data for HIV viral load, the LAg-Avidity assay, or the JHU BioRad-Avidity assay; one seroconverter was excluded because of missing viral load data. The final study cohort included 220 seroconverters and 4,396 non-seroconverters; 3,497 (79.5%) of the non-seroconverters were known to be infected >2 years. Viral load was ≤400 copies/mL at the PC24 visit for 63 (28.6%) of the seroconverters and 3,226 (73.4%) of the non-seroconverters. Datasets for the final seroconverter and non-seroconverter groups are provided (S1 and S2 Datasets, respectively).

### Performance of MAAs for identifying persons with recent HIV infection

We evaluated the performance of four MAAs: (1) the MAA recommended by the US CDC that includes the LAg-Avidity assay and viral load (LAg MAA) [15, 22, 24]; (2) a MAA optimized for incidence estimation in populations with subtype C HIV that includes the LAg-Avidity assay, JHU BioRad-Avidity assay, and viral load (MAA-C) [9]; (3) a MAA that includes the rapid LAg assay and viral load (Rapid MAA) [23, 41], and a MAA that includes the LAg-Avidity and JHU BioRad-Avidity assays without viral load (Alternate MAA [4]) (Table 1). The mean window period for a MAA indicates the average time after infection that individuals are classified as recent. The recommended mean window periods for the LAg MAA, MAA-C, Rapid MAA, and Alternate MAA are 130 days [22], 248 days [9], 180 days [23], and 119 days [4], respectively (Table 1).

As a first step, each MAA was assessed for the ability to identify persons infected <1 year as recently infected (Table 2). The four MAAs classified different numbers of the 220 seroconverters as recent (LAg MAA: 60 [27%]; MAA-C: 100 [45%]; Rapid MAA: 54 [25%]; Alternate MAA: 81 [37%]). The number of seroconverters classified as recent by each MAA was not proportional to the mean window period. For example, the MAA with the shortest mean window period (Alternate MAA) classified more seroconverters as recent than two MAAs that had longer mean window periods (the LAg and Rapid MAAs). The MAA-C has the longest mean window period and classified the highest number of seroconverters as recent; the number of seroconverters identified as recent was only about 20% higher than the number classified as recent by the Alternate MAA, even though the window period was more than twice as long.

**Table 2:** Performance of multi-assay algorithms for identifying persons infected less than one year as recently infected.

	Seroconverters (N = 220)		Non-seroconverters (N = 4,396)						
MAA	True recent	False non-recent	True non-recent	False recent	Sensitivity	Specificity	Positive predictive value	Negative predictive value	False recent rate
LAg MAA	60	160	4,385	11	27.3%	99.7%	84.5%	96.5%	0.2%
MAA-C	100	120	4,362	34	45.5%	99.2%	74.6%	97.3%	0.5%
Rapid MAA	54	166	4,379	17	24.5%	99.6%	76.1%	96.3%	0.4%
Alternate MAA	81	139	4,336	69^a^	36.8%	98.4%	54.0%	96.9%	1.3%

The table shows the performance characteristics for four multi-assay algorithms (MAAs; see Table 1). The cohort included 220 persons who were infected for less than one year (seroconverters) and 4,396 persons who were infected for more than one year (non-seroconverters). True recent (TR) indicates the number of seroconverters classified as recently infected; false non-recent (FNR) indicates the number of seroconverters classified as not recently infected. True non-recent (TNR) indicates the number of non-seroconverters classified as not recently infected; false recent (FR) indicates the number non-seroconverters classified as recently infected. For sensitivity and specificity calculations, true positive results were cases where the MAA identified persons infected <1 year as recently infected. Sensitivity was calculated as TR/(TR+FNR). Specificity was calculated as TNR/(TNR+FR). Positive predictive value was calculated as TR/(TR+FR). Negative predictive value was calculated as TNR/(TNR+FNR). The false recent rate (FRR) was calculated as the percentage of samples from the 3,497 persons analyzed who were infected more than two years who were misclassified as recently infected.

^a^Sixty (87.0%) of these individuals were virally suppressed (VL ≤ 400 copies/mL).

Abbreviations: MAA: multi-assay algorithm, LAg: limiting antigen assay, VL: viral load.

The four MAAs also misclassified different numbers of the 4,396 non-seroconverters as recent (LAg MAA: 11 [0.3%]; MAA-C: 34 [0.8%]; Rapid MAA: 17 [0.4%]; Alternate MAA: 69 [1.6%]). The majority of the non-seroconverters that were misclassified as recent by the Alternate MAA had viral loads <400 copies/mL (60/69 [87%]). The FRR (percentage of persons infected >2 years who were classified as recent) for the LAg MAA, MAA-C, Rapid MAA, and Alternate MAA was 0.2%, 0.5%, 0.4%, 1.3%, respectively.

### Subgroups of seroconverters identified as recent vs. non-recent using different MAAs

**Fig 2:**
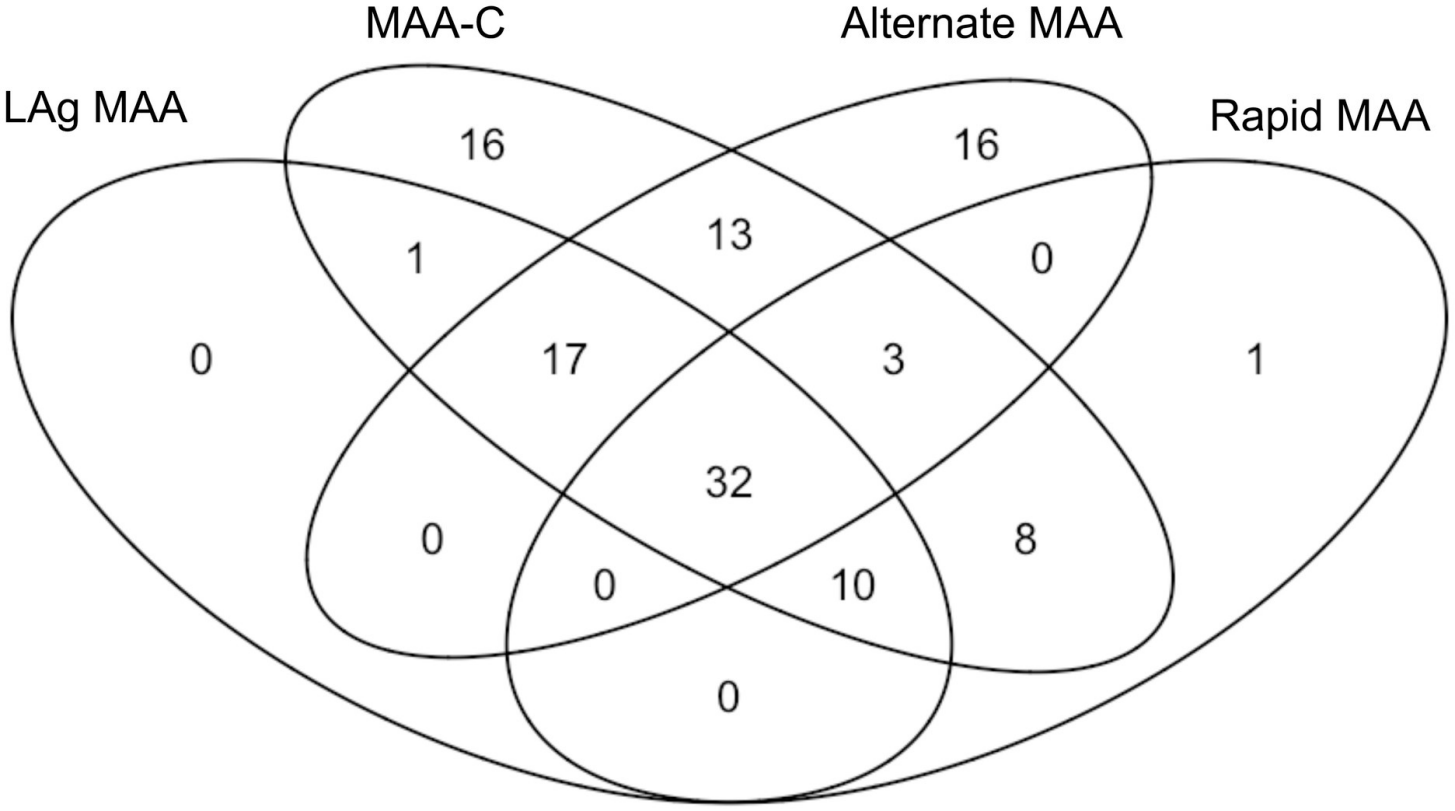
Comparison of the subsets of seroconverters identified as recently infected using four MAAs. The Venn diagram shows the number of seroconverters who were classified as recently infected using one or more of the four MAAs. Thirty-two of the 220 seroconverters (15%) were classified as recently infected by all four MAAs. Abbreviations: LAg: limiting antigen assay, MAA: multi-assay algorithm.

We compared the groups of individuals that were classified as recent vs. non-recent using the four MAAs. Only 32 (15%) of the 220 seroconverters were classified as recent by all four MAAs (Fig 2). More detailed analysis was performed for the three MAAs that had lower FRRs (S1 and S2 Figs). We next compared testing outcomes for the LAg MAA and MAA-C (S1 Fig). The recent group identified by the LAg MAA was a subset of the recent group identified by the MAA-C. In contrast, different, overlapping groups of non-seroconverters were misclassified as recent by these two MAAs. We also compared the testing outcomes for the two LAg-based MAAs (LAg-Avidity assay plus viral load; Rapid LAg assay plus viral load) (S2 Fig). Different, overlapping groups of seroconverters and non-seroconverters were classified as recent using these two MAAs. If technical variability near the 1.5 OD-n assay cutoff were responsible for differences in recency outcome, the LAg values for participants classified as recent by only one of the two MAAs should be close to this cutoff. However, this was not the case. The LAg-Avidity assay values for the seroconverters who were classified as recent by only one of the two MAAs ranged from 0.42 to 2.83 OD-n, and values for non-seroconverters ranged from 0.65 to 4.43 OD-n (S2 Fig).

### Evaluation of assay performance for seroconverters with ART-induced viral suppression

As noted above, viral suppression from ART can lead to misclassification of persons with long-standing infection as recent when using MAAs that do not contain viral load as a biomarker; this most likely reflects down-regulation of anti-HIV antibodies. We next evaluated whether ART-induced viral suppression also impacts results obtained for seroconverters using serologic incidence assays. We compared the results obtained using the LAg-Avidity assay and JHU BioRad-Avidity assay in two participants groups: viremic seroconverters (VL>400 copies/mL; n = 157; five had ARV drugs detected, five did not have ARV drug data) and seroconverters with ART-induced viral suppression (VL≤400 copies/mL + ARV drugs detected; n = 49) (Table 3).

**Table 3:** Serologic assay results for viremic seroconverters vs. seroconverters who were virally suppressed on ART.

Characteristic	Viremic^a^ (N = 157)	Suppressed, on ART^b^ (N = 49)	Univariate P-value	Multivariate P-value
**LAg-Avidity, mean OD-n (SD)**	2.19 (1.47)	1.96 (1.76)	0.409	
**LAg-Avidity <1.5**			**0.0096**	**0.0106**
**Yes**	63 (40%)	30 (61%)		
**No**	94 (60%)	19 (39%)		
**Rapid LAg, no long-term band**			**0.00047**	**0.0003**
**No**	102 (65%)	18 (37%)		
**Yes**	55 (35%)	31 (63%)		
**BioRad AI, mean % (SD)**	61 (31)	71 (26)	**0.021**	**0.033**
**BioRad AI <95%**			0.23	
**Yes**	127 (81%)	36 (73%)		
**No**	30 (19%)	13 (27%)		

The table compares results obtained with the LAg-Avidity assay, Rapid Lag assay, and BioRad-Avidity assay for viremic seroconverters and seroconverters who were virally suppressed on ART.

^a^ Viral suppression was defined as having a viral load ≤400 copies/mL.

^b^ Laboratory detection of one or more ARV drugs was considered to be indicative of ART.

Abbreviations: AI: avidity index, ART: antiretroviral therapy, VL: viral load.

The mean OD-n value obtained for these two groups was similar using the LAg-Avidity assay (viremic: 2.19 OD-n [standard deviation (SD): 1.47]; suppressed on ART: 1.96 OD-n [SD: 1.76]; p = 0.409). However, seroconverters who were suppressed on ART were more likely to have LAg-Avidity values below the cutoff used in the LAg MAA (<1.5 OD-n) than those who were viremic (30/49 [61%] vs. 63/157 [40%], p = 0.0096). Seroconverters who were suppressed on ART were also less likely to have the long-term infected band on the rapid LAg assay (18/49 [37%] vs. 102/157 [65%], p = 0.00047). The mean JHU BioRad-Avidity value was significantly higher in the ART-suppressed seroconverters than in those who were not suppressed (71% [SD: 26] vs. 61% [SD: 31], p = 0.021); this is consistent with the expected longer duration of HIV infection in those who were suppressed on ART by the PC24 visit. However, there was no difference between groups for the proportion of seroconverters who had a JHU BioRad-Avidity assay result <95% (the cutoff used in the MAA-C; suppressed on ART: 36/49 [73%], viremic: 127/157 [81%]; p = 0.23).

The associations noted above were also observed in multivariate models that included viral suppression status, sex, age, country, and study arm as input variables. Viral suppression on ART was independently associated with having a LAg OD-n value <1.5 (p = 0.0106; odds ratio [OR]: 2.36; 95% confidence intervals [CI]: 1.22, 4.55; viral suppression status was the only variable in this model after backwards selection). Viral suppression on ART was also independently associated with not having the long-term infected band on the rapid LAg assay (p = 0.0003; adjusted odds ratio [aOR]: 3.60; 95% CI: 1.77, 7.30; the final backward-selected model also included sex, country and study arm). In contrast, viral suppression on ART was independently associated with having a higher mean JHU BioRad-Avidity value (p = 0.033; difference in means: 10.5%, CI: 0.90%, 20.05%; viral suppression status was the only variable in this model after backwards selection); higher JHU BioRad-Avidity values are usually associated with longer duration of infection.

### Comparison of assay performance for seroconverters with natural vs. ART-induced viral suppression

We next compared results obtained with the LAg-Avidity and JHU BioRad-Avidity assays using samples from seroconverters with natural vs. ART-induced viral suppression (Table 4); samples were classified into these two groups based on results from the ARV drug assay. Participant groups included seroconverters with ART-induced viral suppression (n = 49, same group used in the analysis above) and virally-suppressed seroconverters with no ARV drugs detected (n = 13; one suppressed participant did not have ARV drug data and was excluded). There was no statistical difference in the mean values obtained for the LAg-Avidity-Assay (suppressed on ART: 1.96 OD-n [SD: 1.76], no ARV drugs detected: 2.37 OD-n [SD: 1.51]; p = 0.406) or the JHU BioRad-Avidity assay (suppressed on ART: 71% [SD: 26], no ARV drugs detected: 72% [SD: 28]; p = 0.94) for these two groups. There was also no statistical difference between the two groups for the proportion of persons with a JHU BioRad-Avidity assay result below the cutoff used in the MAA-C (95%) (suppressed on ART: 36/49 [73%], no ARV drugs detected: 9/13 [69%]; p = 0.74) or the proportion of persons with no long-term band detected using the rapid LAg assay (suppressed on ART: 31/49 [63%], no ARV drugs detected: 6/13 [46%]; p = 0.264). However, seroconverters who were suppressed on ART were more likely to have LAg-Avidity values below the cutoff used in the LAg MAA (<1.5 OD-n) than those who had no ARV drugs detected (30/49 [61%] vs. 3/13 [23%], p = 0.014). In a multivariate model that included viral suppression type (natural vs. ART-induced), sex, age, country, and study arm as input variables, ART-induced viral suppression was independently associated with having a LAg OD-n value <1.5 (p = 0.014; aOR: 6.44, CI: 1.47, 28.3; the final backward-selected model also included country).

**Table 4:** Serologic assay results for seroconverters who were virally suppressed on ART vs. seroconverters with natural viral suppression.

Characteristic	Suppressed, on ART^a^^,^ ^b^ (N = 49)	Suppressed, no ARV detected (N = 13)	P-value
**LAg-Avidity, mean OD-n (SD)**	1.96 (1.76)	2.37 (1.51)	0.406
**LAg-Avidity <1.5**			**0.014**
**Yes**	30 (61%)	3 (23%)	
**No**	19 (39%)	10 (77%)	
**Rapid LAg, no long-term band**			0.264
**No**	18 (37%)	6 (46%)	
**Yes**	31 (63%)	7 (54%)	
**BioRad AI, mean % (SD)**	71 (26)	72 (28)	0.94
**BioRad AI <95%**			0.74
**Yes**	36 (73%)	9 (69%)	
**No**	13 (27%)	4 (31%)	

The table compares results obtained with the LAg-Avidity assay, Rapid Lag assay, and BioRad-Avidity assay for seroconverters who were virally suppressed on ART vs. seroconverters with natural viral suppression.

^a^ Viral suppression was defined as having a viral load ≤400 copies/mL.

^b^ Laboratory detection of one or more ARV drugs was considered to be indicative of ART.

Abbreviations: AI: avidity index, ART: antiretroviral therapy, VL: viral load.

## Discussion

This study compared the performance of four MAAs for individual-level recency assessments. These MAAs were developed for population-level HIV incidence estimation and were not optimized for individual recency assessments. All four MAAs classified <50% of the seroconverters as recent (range 24% to 45%), and only 15% of the seroconverters were classified as recent by all four MAAs. The length of the window period did not correlate with the number of seroconverters classified as recent. The MAA-C, which has the longest mean window period of the four MAAs, identified the highest number of seroconverters as recent. In contrast, the Alternate MAA, which has the shortest mean window period, classified more seroconverters as recently infected than two other MAAs. The FRR also varied for the four MAAs (from 0.2% to 1.3%). The two MAAs that classified the highest number of seroconverters as recent (the MAA-C and Alternate MAA) also had the highest FRRs. This illustrates the trade-off between identifying more recently-infected individuals and misclassifying more individuals with long-term infection as recently infected with some MAAs. In this study, the Rapid MAA classified the lowest number of seroconverters as recent, despite having a relatively high mean window period. Analysis of this MAA by the Consortium for the Evaluation and Performance of HIV Incidence Assays (CEPHIA) suggested that the mean window period for this MAA may be lower than the window period provided by the manufacturer [41], which could explain our findings.

We also found that the MAAs identified different subsets of seroconverters as recently infected. This was even observed for the LAg MAA and Rapid MAA; both of these MAAs include a LAg-based assay with the same target antigen and use the same viral load cutoff. Further research is needed to understand performance differences between the laboratory-based and point-of-care LAg assays.

ART-induced viral suppression impacted the performance of the LAg-Avidity and rapid LAg assays, but did not meaningfully impact the performance of the JHU BioRad-Avidity assay, consistent with prior studies [4, 9]. The effect of viral suppression on the performance of the two LAg-based assays would not impact performance of MAAs that classify all virally-suppressed persons as non-recent. However, it may impact use of these assays in MAAs that do not include viral load. Interestingly, the performance of the LAg-Avidity assay differed in persons with natural vs. ART-induced viral suppression (individuals who were virally suppressed on ART had lower LAg-Avidity values than those who were virally suppressed in the absence of detectable ARV drugs). Further research is needed to understand the differences in serological responses between individuals with early viral suppression on ART and those who have low viral loads in the absence of ART.

One limitation of this study is that the study cohort only included individuals in a population survey from regions with predominantly subtype C HIV infection; results may differ in cohorts with other HIV risk factors and subtypes. Also, because study visits in HPTN 071 (PopART) were conducted annually, we were not able to assess performance of the MAAs for persons who were known to be infected for shorter periods. For sensitivity and specificity calculations, true positive results were defined as cases where the MAA identified persons infected <1 year as recently infected. Of note, all four MAAs have window periods less than a year (119 to 248 days) and would therefore not be expected to identify all persons infected <1 year as recently infected. In addition, as noted previously, the interval between the PC12 and PC24 visits in HPTN 071 (PopART) was not precisely one year. An additional limitation is that we only retested specimens with a LAg-Avidity result <2.0 OD-n, per manufacturer guidelines. These guidelines were developed to ensure assay reliability for a cutoff of 1.5 OD-n. This study included MAAs with a higher cutoff (2.8 OD-n), but guidelines for specimen retesting were not adjusted. Another limitation of this study is that the rapid LAg assay test strips were read manually by a single individual without validation by multiple readers for quality control; the manufacturers now recommend using an electronic reader to interpret test results. Further studies are needed to evaluate performance of the rapid LAg assay and Rapid MAA using manual vs. automated test interpretation.

This study highlights some of the limitations in using MAAs for individual-level assessments of the recency of HIV infection. These limitations should be considered before using MAAs for applications that include informing persons of their test results, since misclassification of the timing of infections could potentially lead to social harms. Further research is needed to develop MAAs that can accurately identify individuals with recent vs. nonrecent HIV infection. This study also demonstrates that the impact of viral load on incidence assays is different for those with natural vs. ART-induced viral suppression. Further research is needed to determine if this impacts performance of MAAs for HIV incidence estimation or other applications.

## Supporting information

S1 Fig Comparison of the subsets of participants identified as recently infected using the LAg MAA and MAA-C. The Venn diagrams show the testing outcomes for the LAg MAA and MAA-C. Panel A shows the number of seroconverters who were classified as recently infected using one or both MAAs (total seroconverters evaluated: 220). Panel B shows the number of non-seroconverters who were misclassified as recently infected using one or both MAAs (total seroconverters evaluated: 4,396). Abbreviations: LAg: limiting antigen assay, MAA: multi-assay algorithm.
(TIF)

S2 Fig Comparison of the subsets of participants identified as recent using the LAg MAA and Rapid MAA. The Venn diagrams show the testing outcomes for the two LAg-based MAAs: the LAg MAA and Rapid MAA. Panel A shows the number of seroconverters who were classified as recently infected using one or both of the MAAs (total seroconverters evaluated: 220). Panel C shows the number of non-seroconverters who were misclassified as recent with one or both MAAs (total non-seroconverters evaluated: 4,396). The graphs show the OD-n values obtained with the LAg-Avidity assay for seroconverters (B) and non-seroconverters (D) who were classified as recently infected using one or both of the MAAs. Abbreviations: LAg: limiting antigen assay, MAA: multi-assay algorithm, OD-n: normalized optical density.
(TIF)

S1 Table HTPN 071 (PopART) study team listing. (DOCX)

S1 Dataset Minimum dataset, seroconverters. (XLSX)

S2 Dataset Minimum dataset, nonseroconverters. (XLSX)
