# Supplementary figures and images for: Evaluation of multi-assay algorithms for identifying individuals with recent HIV infection: HPTN 071 (PopART)
Source: PLoS One. 2021 Dec 17;16(12):e0258644. doi: 10.1371/journal.pone.0258644 (PMC8682874; doi:10.1371/journal.pone.0258644)

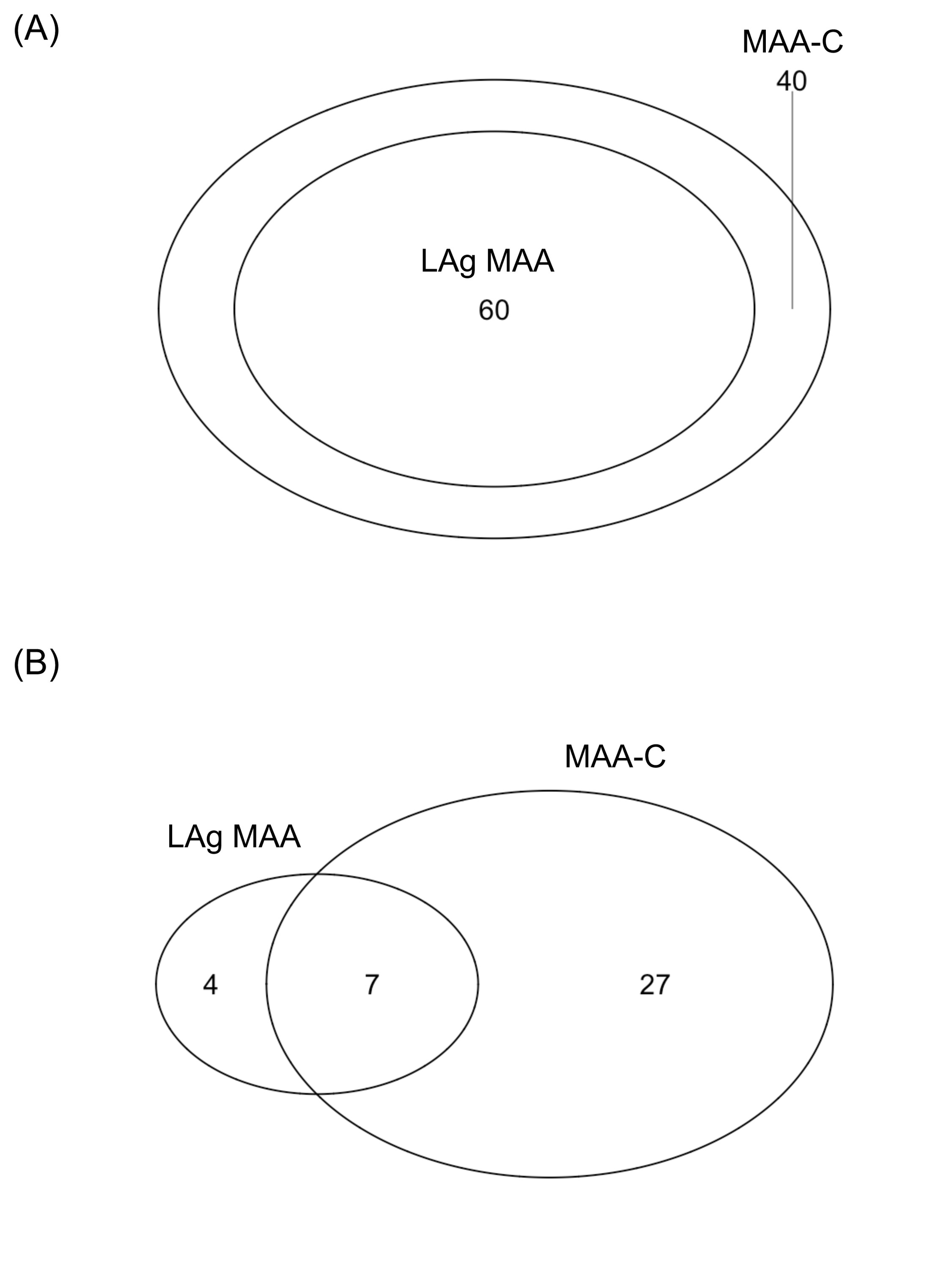

Supplement: S1 Fig — The Venn diagrams show the testing outcomes for the LAg MAA and MAA-C. Panel A shows the number of seroconverters who were classified as recently infected using one or both MAAs (total seroconverters evaluated: 220). Panel B shows the number of non-seroconverters who were misclassified as recently infected using one or both MAAs (total seroconverters evaluated: 4,396). Abbreviations: LAg: limiting antigen assay, MAA: multi-assay algorithm. (TIF) [file pone.0258644.s001.tif]

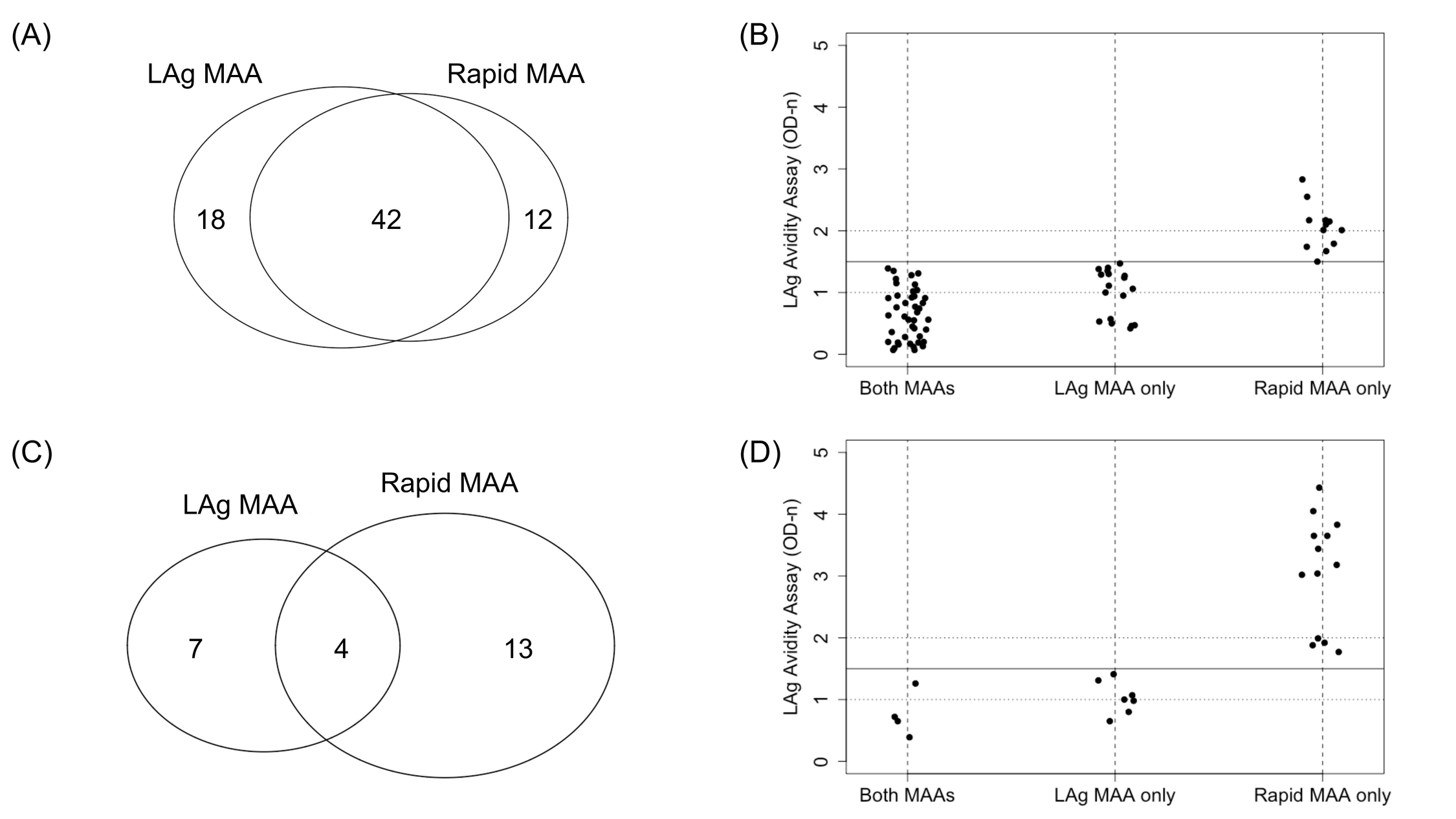

Supplement: S2 Fig — The Venn diagrams show the testing outcomes for the two LAg-based MAAs: the LAg MAA and Rapid MAA. Panel A shows the number of seroconverters who were classified as recently infected using one or both of the MAAs (total seroconverters evaluated: 220). Panel C shows the number of non-seroconverters who were misclassified as recent with one or both MAAs (total non-seroconverters evaluated: 4,396). The graphs show the OD-n values obtained with the LAg-Avidity assay for seroconverters (B) and non-seroconverters (D) who were classified as recently infected using one or both of the MAAs. Abbreviations: LAg: limiting antigen assay, MAA: multi-assay algorithm, OD-n: normalized optical density. (TIF) [file pone.0258644.s002.tif]
