# Supplementary material for: Evaluation of multi-assay algorithms for identifying individuals with recent HIV infection: HPTN 071 (PopART)
Source: PLoS One. 2021 Dec 17;16(12):e0258644. doi: 10.1371/journal.pone.0258644 (PMC8682874; doi:10.1371/journal.pone.0258644)
Supplement: S1 Table — (DOCX) [file pone.0258644.s003.docx]

**S1 Table. HTPN 071 (PopART) Study Team listing.**

| **Name** | **South Africa** | **Zambia** | **UK** | **US** | **Affiliation** | **Role** |
| --- | --- | --- | --- | --- | --- | --- |
| Richard Hayes^a^ |  |  | ✓ |  | London School of Hygiene and Tropical Medicine (LSHTM) | Study PI |
| Sarah Fidler |  |  | ✓ |  | Imperial College | Study co-PI |
| Nulda Beyers | ✓ |  |  |  | Desmond Tutu Tuberculosis Centre | Site PI |
| Helen Ayles |  | ✓ |  |  | ZAMBART | Site PI |
| Peter Bock | ✓ |  |  |  | Desmond Tutu Tuberculosis Centre | Site co-PI |
| Wafaa El-Sadr |  |  |  | ✓ | HIV Prevention Trials Network (HPTN) Leadership and Operations Center (LOC) | Network Co-PI |
| Myron Cohen |  |  |  | ✓ | HPTN LOC | Network Co-PI |
| Susan Eshleman |  |  |  | ✓ | HPTN Laboratory Center (LC) | LC PI |
| Yaw Agyei |  |  |  | ✓ | HPTN LC | Senior International Laboratory QA/QC Coordinator |
| Estelle Piwowar-Manning |  |  |  | ✓ | HPTN LC | Deputy Director |
| Virginia Bond |  | ✓ |  |  | ZAMBART | Social Scientist |
| Graeme Hoddinott | ✓ |  |  |  | Desmond Tutu Tuberculosis Centre | Social Scientist |
| Deborah Donnell |  |  |  | ✓ | HPTN Statistical and Data Management Center (SDMC) | SDMC PI, Statistician |
| Sian Floyd |  |  | ✓ |  | LSHTM | Statistician |
| Ethan Wilson |  |  |  | ✓ | HPTN SDMC | Statistician |
| Lynda Emel |  |  |  | ✓ | HPTN SDMC | Data Management |
| Heather Noble |  |  |  | ✓ | HPTN SDMC | Clinical Data Manager |
| Dave McLeod |  |  | ✓ |  | LSHTM | Statistician |
| David Burns |  |  |  | ✓ | NIAID | Medical Officer |
| Christophe Fraser |  |  | ✓ |  | Oxford University | Mathematical Modeler |
| Anne Cori |  |  | ✓ |  | Imperial College | Mathematical Modeler |
| Nirupama Deshmane Sista |  |  |  | ✓ | HPTN LOC | HPTN, Director |
| Sam Griffith |  |  |  | ✓ | HPTN LOC | Senior Clinical Research Manager |
| Ayana Moore |  |  |  | ✓ | HPTN LOC | Scientist |
| Tanette Headen |  |  |  | ✓ | HPTN LOC | Prevention Research Specialist |
| Rhonda White |  |  |  | ✓ | HPTN LOC | Senior Community Program Manager |
| Eric Miller |  |  |  | ✓ | HPTN LOC | Communications Manager |
| James Hargreaves |  |  | ✓ |  | LSHTM | Epidemiologist |
| Katharina Hauck |  |  | ✓ |  | Imperial College | Health Economist |
| Ranjeeta Thomas |  |  | ✓ |  | Imperial College | Health Economist |
| Mohammed Limbada |  | ✓ |  |  | ZAMBART | Clinical Scientist |
| Justin Bwalya |  | ✓ |  |  | ZAMBART | Population Cohort Manager |
| Michael Pickles |  |  | ✓ |  | University of Manitoba | Mathematical Modeler |
| Kalpana Sabapathy |  |  | ✓ |  | LSHTM | Clinical Epidemiologist |
| Ab Schaap |  | ✓ |  |  | ZAMBART | Senior Data Manager |
| Rory Dunbar | ✓ |  |  |  | Desmond Tutu Tuberculosis Centre | Senior Data Manager |
| Kwame Shanaube |  | ✓ |  |  | ZAMBART | Site coordinator, Principal Investigator adolescent study |
| Blia Yang | ✓ |  |  |  | Desmond Tutu Tuberculosis Centre | Intervention manager |
| Musonda Simwinga |  | ✓ |  |  | ZAMBART | Community Engagement Coordinator |
| Peter C. Smith |  |  | ✓ |  | Imperial College Business School | Emeritus Professor of Health Policy |
| Sten Vermund |  |  |  | ✓ | HPTN | HPTN Executive Committee Representative |
| Nomtha Mandla | ✓ |  |  |  | Desmond Tutu Tuberculosis Centre | Population Cohort Manager |
| Nozizwe Makola | ✓ |  |  |  | Desmond Tutu Tuberculosis Centre | Community Coordinator |
| Anneen van Deventer | ✓ |  |  |  | Desmond Tutu Tuberculosis Centre | Laboratory Manager |
| Anelet James | ✓ |  |  |  | Desmond Tutu Tuberculosis Centre | Laboratory Manager |
| Karen Jennings | ✓ |  |  |  | City Health Department, City of Cape Town | Head of HIV/TB Program |
| James Kruger | ✓ |  |  |  | Department of Health, Western Cape | Director of HIV, AIDS, STI & TB |
| Mwelwa Phiri |  | ✓ |  |  | ZAMBART | Intervention Manager |
| Barry Kosloff |  | ✓ |  |  | ZAMBART | Zambian Lab Manager |
| Lawrence Mwenge |  | ✓ |  |  | ZAMBART | Health Economist |
| Sarah Kanema |  | ✓ |  |  | ZAMBART | Health Economist |
| Rafael Sauter |  |  | ✓ |  | Oxford University | Mathematical Modeler |
| Will Probert |  |  | ✓ |  | Oxford University | Mathematical Modeler |
| Ramya Kumar |  | ✓ |  |  | ZAMBART | Epidemiologist |
| Ephraim Sakala |  | ✓ |  |  | ZAMBART | Intervention Manager |
| Andrew Silumesi |  | ✓ |  |  | Ministry of Health, Zambia | Director of Research |
| Tim Skalland |  |  |  | ✓ | HPTN SDMC | Statistician |
| Krista Yuhas |  |  |  | ✓ | HPTN SDMC | Statistician |

^a^ Email: Richard.Hayes@lshtm.ac.uk
